# Racial and socioeconomic disparities in patients with Waldenström Macroglobulinemia: a SEER database analysis

**DOI:** 10.1038/s41598-026-54387-2

**Published:** 2026-06-03

**Authors:** Alvaro Alvarez Soto, Junmin Song, Maxime Braun, Swarup Kumar

**Affiliations:** 1 Carole and Ray Neag Comprehensive Cancer Center, University of Connecticut Health (UCONN Health), Farmington, CT USA; 2 Hematology/Oncology, Brown University Health, Providence, United States; 3 Department of Medicine, Jacobi Medical Center, Albert Einstein College of Medicine, Bronx, NY USA; 4 University of Connecticut School of Medicine (UCONN Health), Farmington, CT USA

**Keywords:** Oncology, Cancer epidemiology, Cancer

## Abstract

Waldenström Macroglobulinemia (WM) is a rare plasma cell disorder with a prolonged smoldering phase, and treatment initiation is typically delayed. While multiple therapeutic options exist for WM, it remains unclear whether minoritized and low socioeconomic status (SES) patients experience equal or similar survival outcomes. We conducted a retrospective study using the SEER database, identifying WM patients diagnosed between 2006 and 2020. We examined overall survival (OS) and cancer-specific survival (CSS) disparities in 8256 WM patients by race/ethnicity and SES, as defined by the Yost index. Race/ethnicity was categorized as Non-Hispanic White (NHW), Non-Hispanic Black (NHB), Hispanic, and Non-Hispanic Other. Logistic regression showed that lower SES was associated with greater chemotherapy use (OR = 1.33, 95% CI, 1.22–1.46). Low SES was linked to increased all-cause mortality (HR = 1.37, 95% CI, 1.27–1.47). In stratified analyses, low SES was associated with worse OS in NHW and Hispanic patients but not in NHB patients. No differences in CSS were seen between low and high SES groups in the univariate Cox regression. However, after adjustment and competing risk analysis low SES patients had worse CSS. These findings suggest that SES is associated with survival disparities in WM, underscoring the critical role of addressing social determinants of health to ensure equitable outcomes.

**Supplementary Information:**

The online version contains supplementary material available at 10.1038/s41598-026-54387-2.

## Background

Waldenström Macroglobulinemia (WM) is a type of plasma cell disorder characterized by the presence of monoclonal IgM protein and ≥ 10% lymphoplasmacytic cells in the bone marrow^1^. WM typically has a prolonged smoldering phase, and treatment is frequently delayed until indicated based on clinical and laboratory criteria^2^. Survival outcomes for WM have improved in the last years^3^, possibly due to the optimization of chemoimmunotherapy regimens and the introduction of Bruton’s Tyrosine Kinase (BTK) inhibitors^4–6^. While those interventions have likely improved WM survival trends, it is unclear whether minoritized populations and patients with low socioeconomic status (SES) patients have equally benefited from these advancements.

Previous population-based studies have looked into WM outcomes by race, but the results are conflicting, with some studies showing worse survival among Blacks^7^ or Hispanics^8^, and others finding no significant racial differences in outcomes^9^. While these studies have deepened our understanding of racial disparities in WM outcomes, they have not accounted for the impact of SES. In other malignancies, adjusting for SES has been shown to reduce survival disparities in many, though not all, cancer types^10^. In WM, understanding whether survival differences are driven by race alone, SES alone, or a combination of both is crucial, as it can help guide efforts to investigate biological versus environmental factors.

Given the lack of studies examining the intersection of social factors and race in WM, we have designed a population-based study to investigate disparities in overall survival (OS) and cancer-specific survival (CSS) among patients with WM based on race and SES. We believe both variables should be assessed together when examining outcome differences (OS and CSS). As a secondary objective, we aim to investigate differences in the use of systemic treatment based on race/ethnicity and SES.

## Methods

We conducted a retrospective, population-based study using the Surveillance, Epidemiology, and End Results (SEER) database. We utilized the SEER software case listing session to compile a matrix of all patients diagnosed with Waldenstrom macroglobulinemia (ICD-O-3 code 9761/3) available in the dataset. Patients with gamma heavy chain disease were excluded as this is considered a different type of lymphoplasmacytic lymphoma. The patients in our dataset were diagnosed between 2006 and 2020. We also used the SEER Research Plus Data, 18 Registries, Nov 2020 submission linked to country attributes (Database ID = 2135). Permission was obtained from the SEER to use this specialized database, which is linked to county-level socioeconomic status (SES) using the Yost index. We used the STROBE guidelines to enhance the quality of reporting this observational study^11^. Before initiating this study, a request was submitted to the University of Connecticut Institutional Review Board (IRB), and it was deemed exempt as it uses de-identified, publicly available data.

### Variables

Self-reported race/ethnicity data is available in the SEER database and was grouped into 4 categories: Non-Hispanic Black (NHB), Non-Hispanic White (NHW), Hispanics, and Non-Hispanic of other races (NH-others). To assess SES, we utilized the Yost index, which is provided by the SEER database. This index incorporates census-level data, including median household income, median house value, median gross rent, the percentage of individuals living below 150% of the poverty line, an education index, the percentage of working-class individuals, and the percentage of unemployed individuals. The Yost index is divided into five quintiles, with Yost quintile 1 corresponding to the lowest SES and Yost quintile 5 to the highest SES. The association between the Yost index and outcomes has been validated in multiple solid malignancies and multiple myeloma^12–14^. Additional demographics including age, sex, and marital status, were also collected. We collected data on overall survival (OS) time and cancer-specific survival (CSS) time. OS is defined as time (in months) from diagnosis to death of any cause, and CSS as time (in months) from diagnosis to WM death. Other variables included chemotherapy use (yes or no) and radiation therapy use and type.

### Statistical analysis

We compared the baseline characteristics of the high SES group (YOST 4–5) and the low SES group (YOST 1–3) based on demographics, marital status, stage, and therapy received. Group comparisons were conducted using Chi-square tests. To assess differences in receiving chemotherapy among race and ethnic groups, we performed logistic regression and calculated odds ratios (ORs). For OS and CSS, we conducted survival analyses using the log-rank test and, and univariate and multivariate Cox proportional hazards regression, these are considered the primary statistical analyses for survival outcomes. In addition, cumulative incidence analyses with Fine–Gray subdistribution hazard models were performed to account for competing risks of non-cancer mortality in CSS. A two-sided p-value < 0.05 was considered statistically significant. Missing data were removed from the analyses. All analyses were conducted using R software (version 4.4.0) and its associated packages *(*https://www.r-project.org/).

## Results

We found 8256 patients with a WM diagnosis between 2006 and 2020. In the total population, there were 4992 male patients and 3264 females; regarding race, there were 6976 NHW and 1280 non-White patients. When looking into differences between cohorts, the proportion of male and female patients was similar between the high and low SES cohorts. Patients in the high SES group were more likely to be married as compared to the low SES group (54.9% vs. 43.9%, *p* < 0.001). Differences in race/ethnicity by SES were seen; patients in the high SES cohort were more frequently NHW (88% vs. 78.1%) and less frequently NHB (2.4% vs. 7.7%) or Hispanic (3.9% vs. 9.9%) Regarding treatment differences, chemotherapy was used less frequently in the high SES group (40.6% vs. 48.1%) (Table 1).

**Table 1:** Baseline Characteristics.

	High SES (YOST 4–5)	Low SES (YOST 1–3)	*p*-value
	5069	3187	
Age group			0.997
<65 years	1425 (28.1%)	895 (28.1%)	
≥65 years	3644 (71.9%)	2292 (71.9%)	
Sex			0.11
Male	3100 (61.2%)	1892 (59.4%)	
Female	1969 (38.8%)	1295 (40.6%)	
Race			< 0.001
Hispanic (All Races)	199 (3.9%)	314 (9.9%)	
Non-Hispanic American Indian/Alaska Native	8 (0.2%)	14 (0.4%)	
Non-Hispanic Asian or Pacific Islander	238 (4.7%)	81 (2.5%)	
Non-Hispanic Black	121 (2.4%)	245 (7.7%)	
Non-Hispanic Unknown Race	43 (0.8%)	17 (0.5%)	
Non-Hispanic White	4460 (88.0%)	2516 (78.9%)	
Chemotherapy received (%)			< 0.001
Yes	2058 (40.6%)	1532 (48.1%)	
No	3011 (59.4%)	1655 (51.9%)	
Radiation therapy received (%)			< 0.001
Beam radiation	23 (0.5%)	4 (0.1%)	
No	4536 (89.5%)	2595 (81.4%)	
Not available (IL or TX)	506 (10.0%)	585 (18.4%)	
Recommended, unknown if administered	3 (0.1%)	2 (0.1%)	
Refused	1 (0.0%)	1 (0.0%)	
Marital status at diagnosis (%)			< 0.001
Married	2782 (54.9%)	1398 (43.9%)	
Unmarried	1398 (27.6%)	1005 (31.5%)	
Unknown	889 (17.5%)	784 (24.6%)	

### Treatment differences by SES status

**Fig. 1:**
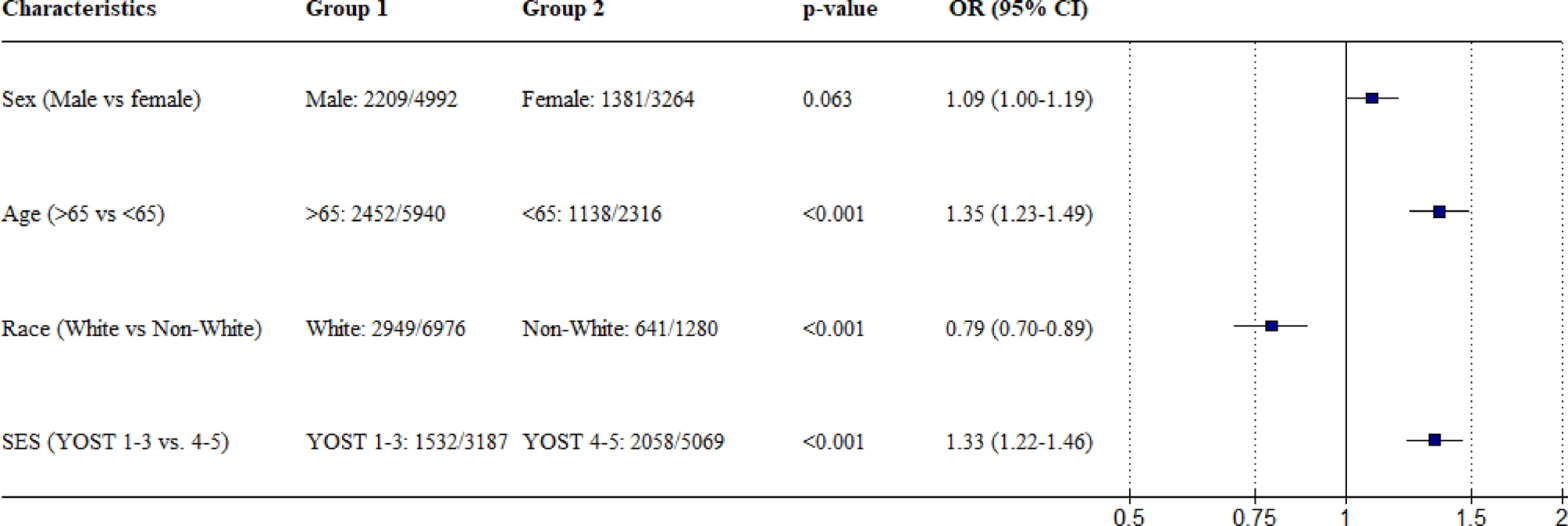
Odds of chemotherapy use by groups. Multivariable logistic regression analysis of chemotherapy use by group. Chemotherapy use (Yes vs. No) was the outcome variable, and the listed characteristics were included as explanatory variables. Values represent the number of patients who received chemotherapy over the total number of patients within each subgroup.

One of the goals of this study was to assess the odds of chemotherapy use by SES and race. We found that younger age was linked with higher odds of chemotherapy use (age < 65 years; OR = 1.35, 95% CI, 1.23–1.49). Low SES was associated with increased odds of chemotherapy use (OR = 1.33, 95% CI, 1.22–1.46). NHW race was associated with lower odds of chemotherapy use (OR = 0.79, 95% CI, 0.70–0.89) (Fig. 1).

### Survival analysis

**Fig. 2:**
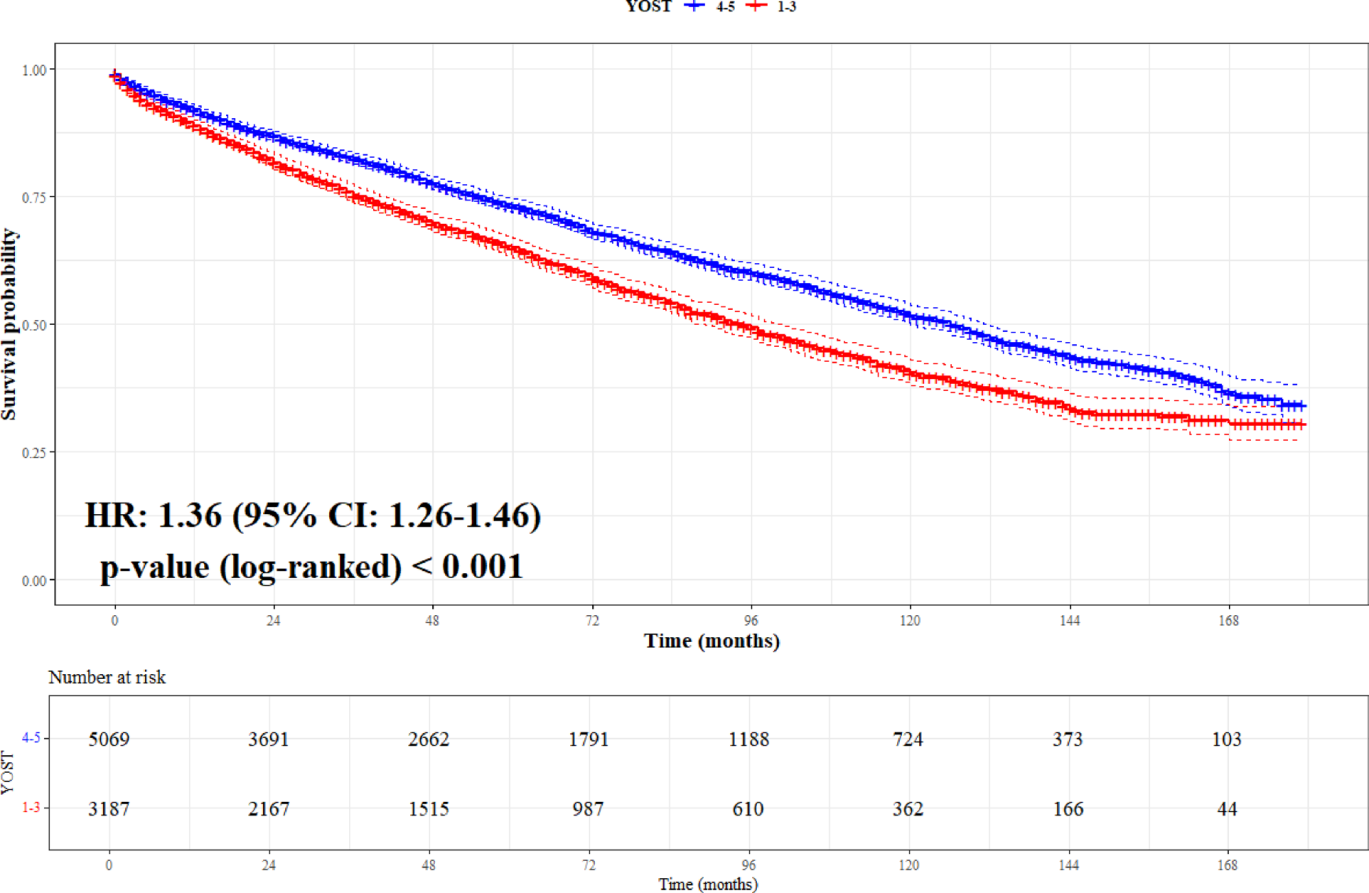
Impact of SES (YOST 4–5 vs. 1–3) in WM in OS among patients with WM. *HR* hazard ratios, *OS* overall survival.

**Fig. 3:**
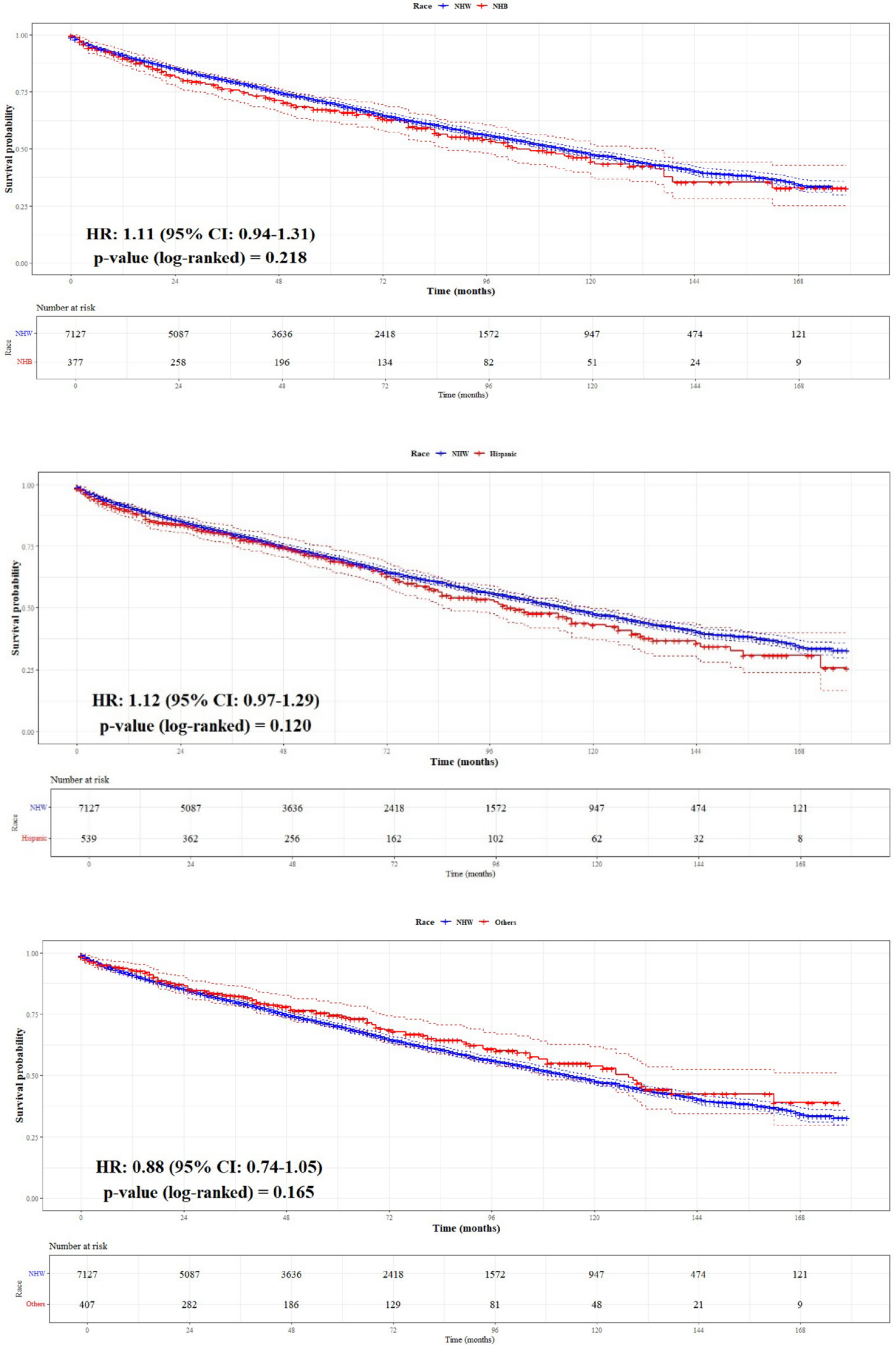
OS stratified by race for patients with WM. From top to bottom; NHW vs. NHB, NHW vs. Hispanics, NHW vs. NH-others. *HR* Hazard ratio.

**Fig. 4:**
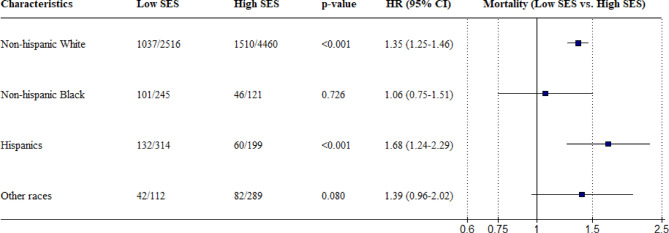
Cox Analysis for all-cause mortality by race and SES (univariate). The low SES group had worse all-cause mortality compared to the high SES group. Values represent the number of patients who passed away over the total number of patients within each subgroup. *CI* Confidence Interval, *HR* Hazard Ratio, *SES* socioeconomic status.

The median follow-up time was 75 months, and the median overall survival was 114 months. Patients in the low SES group had a significantly increased all-cause mortality risk compared with the high SES group (HR = 1.36, 95% CI, 1.26–1.46, *p* < 0.001) (Fig. 2). After adjustment for sex, race, and age low SES was still associated with worse overall survival (HR = 1.36, 95% CI, 1.25–1.46, *p* < 0.001). When investigating all-cause mortality between races, no significant differences were observed between NHW vs. NHB (HR = 1.11, 95% CI, 0.94–1.31), NHW vs. Hispanics (HR = 1.12, 95% CI, 0.97–1.29), or NHW vs. NH-others (HR = 0.88, 95% CI, 0.74–1.05) (Fig. 3). Additional analyses examining racial and ethnic differences in all-cause mortality stratified by SES showed that NHW (HR = 1.35, 95% CI, 1.25–1.47) and Hispanics (HR = 1.68, 95% CI, 1.22–2.26) living in low SES counties had a higher risk of death and worse OS. However, for NHB patients, SES did not impact all-cause mortality risk (HR = 1.06, 95% CI, 0.75–1.50) (Fig. 4).

When analyzing CSS, no significant differences were found in WM-related deaths between high and low SES groups in univariable analysis (HR = 1.24, 95% CI, 0.76–2.01) (Supplementary Fig. 1). No survival differences were observed between NHW patients and other racial or ethnic groups in the overall cohort: NHW vs. NHB (HR = 1.03, 95% CI, 0.97–1.30), NHW vs. Hispanics (HR = 1.17, 95% CI, 0.96–1.43), and NHW vs. NH-others (HR = 1.07, 95% CI, 0.85–1.34) (Supplementary Fig. 2). When examining racial disparities in CSS stratified by SES, NHW and Hispanic patients living in low SES counties had worse survival probabilities (HR = 1.34, 95% CI, 1.19–1.49 and HR = 1.67, 95% CI, 1.11–2.53, respectively). For NHB patients, residing in low or high SES counties did not affect the risk of WM-related death (HR = 0.96, 95% CI, 0.58–1.59) (Supplementary Fig. 3).

The 5-year OS was 65.0% (95% CI, 63.1–66.9) for low SES vs. 73.2% (95% CI, 71.9–74.6) for high SES. The 5-year CSS cumulative incidence was 18.6% (95% CI, 17.1–20.1) vs. 14.2% (95% CI, 13.2–15.3), respectively. In multivariable competing-risks regression, high SES was associated with a lower sub-distribution hazard of cancer-specific death (Fine–Gray sub-HR = 0.80, 95% CI, 0.72–0.88, *p* < 0.001), and the cause-specific Cox model showed a consistent association after adjustment for covariates (HR = 0.75, 95% CI, 0.68–0.83, *p* < 0.001), indicating that lower SES was independently associated with worse cancer-specific survival.

## Discussion

The primary goal of this study was to assess the influence of race and SES on cancer-specific and overall survival among patients with WM. Our univariate model showed that low SES was linked to a higher risk of death from any cause but not cancer-specific risk of death. Regarding race, we found no racial differences in survival outcomes between groups. When looking into survival differences stratified by SES, we found that NHW and Hispanic patients living in low SES counties had worse survival than their counterparts in high SES counties; this finding was not seen among NHB or NH-others, suggesting that SES impacts survival differently among racial groups.

The current understanding of race and WM remains unclear as prior studies have documented varying results. For instance, Castillo et al. conducted two studies using the SEER database and showed that NHB patients have worse relative survival^7^ and higher risk of death^3^ as compared to NHW counterparts. On the other hand, Ailawadhi et al. did not find survival differences between NHB and NHW while utilizing the same SEER database^8^. Our study included patients diagnosed between 2006 and 2020 and did not detect statistically significant differences in survival between race/ethnicity in the overall cohort. Our results may be due to the fact that our study scrutinized a more recent cohort of patients who may have benefited from newer treatments—such as ibrutinib, which was approved in 2016 for the management of WM^15^.

Rather than race, our results showed that SES is significantly associated with the death risk of any cause in patients with WM. This is unsurprising, as current projections show a continuous increase in the financial burden of cancer care for patients in the United States^16^. The management of patients with hematological malignancies, even when indolent, usually entails multiple office and hospital visits along with expensive anti-cancer drugs. These costs potentially restrict the access of patients with low SES to necessary care. While most patients with WM might be beneficiaries of federal aid through MEDICARE based on age (> 65 years), the ancillary costs, such as transportation or caregiver time, might impact their capacity to receive appropriate treatment and follow-up.

Our results showed that NHB race and low SES were linked to higher odds of receiving systemic chemotherapy treatment. This raises the question of whether NHB and low SES patients might have been diagnosed at more advanced stages of the disease. In other indolent lymphomas/leukemias and other plasma cell disorders, racial differences in the burden of disease at presentation have been reported. In multiple myeloma, follicular lymphoma, and chronic lymphocytic leukemia, studies have shown lower hemoglobin at presentation in Black patients as compared to Whites^17–19^. In WM, it is unclear whether the disease burden at presentation differs between races or SES groups, as current data is lacking.

Our study had limitations. First, this is a retrospective study, and confounding might exist despite controlling for available covariates. Patient-related factors that can lead to confounding include comorbidities; if baseline health is different between individuals living in low and high SES, survival outcomes between groups might be different. Other unmeasured factors that can lead to survival differences include, treatment delay, compliance and access to certain therapies (such as stem cell transplant), IPSS risk score, and molecular differences. Second, in SEER, WM is staged as localized and distant, virtually all cases are distant reflecting results from the bone marrow biopsy used in the diagnosis. Third, SES is an ecological variable, it is calculated from census data corresponding to the subject’s county of residence, which does not necessarily represent the individual subject’s socioeconomic status. Fourth, cause-specific mortality in the SEER is based on death certificates and these are not always accurate— This is a potential explanation for no differences in WM-related deaths were found between high and low SES groups.

## Conclusions

In this retrospective cohort, low SES was associated with decreased OS in patients with WM. In addition low SES is linked to worse cancer-specific outcomes after adjustment for covariates and when using competing-risk mortality analysis. If social determinants of health are truly linked to worse outcomes in WM, it is possible that implementing policies that equalize healthcare delivery in low and high-SES counties could equalize survival between groups.

## Future directions

Database analyses help expand the understanding of socioeconomic factors in rare diseases. Further work expanding the data captured is key. Many biological factors are known to impact WM outcomes, but those are frequently not captured in databases. In recent years, there has been an increase in the use of ancillary genomics and molecular-based testing in hematological malignancies. Incorporating new data is critical because it can clarify the longstanding question of biology vs. environment in cancer outcomes.

If cancer databases, like SEER, included detailed information about baseline comorbidities, including other chronic diseases like hypertension, heart disease, kidney disease, among others, we could analyze how these interact with cancer population. Lastly, socioeconomic status is a complex variable, and for its understanding, additional factors like poor quality food, neighborhood environment (and pollution), social support, systemic racism, housing, and the need for multiple employments should be considered. Expanding the capture of that kind of data could be an important step to ensure that cancer outcomes are similar across all socioeconomic groups.

## Supplementary Information

Below is the link to the electronic supplementary material.

Supplementary material 1 (DOCX 2381.5 kb)

## Data Availability

The Data that support the findings of this study are available from the SEER, but restrictions apply to the availability of these data, which were used under license for the current study, and so are not publicly available. However, the corresponding author (Alvaro Alvarez Soto) can send a de-identified dataset upon reasonable request and with the permission of the SEER.
