# Supplementary material for: Racial and socioeconomic disparities in patients with Waldenström Macroglobulinemia: a SEER database analysis
Source: Sci Rep. 2026 Jun 3;16:25375. doi: 10.1038/s41598-026-54387-2 (PMC13473510; doi:10.1038/s41598-026-54387-2)
Supplement: Supplementary file 1 [file 41598_2026_54387_MOESM1_ESM.docx]

**SUPPLEMENTARY FIGURES**

**Supplementary Figure 1.** Impact of socioeconomic status in CSS among patients with WM.

Supplementary Figure 1. Impact of SES in WM in CSS among patients with WM.

**Supplementary Figure 2.** CSS stratified by race for patients with WM.

Supplementary Figure 2. CSS stratified by race for patients with WM. From top to bottom; NHW vs. NHB, NHW vs. Hispanics, NHW vs. NH-others. Abbreviations: CSS, cancer specific survival; HR, Hazard ratio.

**Supplementary Figure 3. Impact of SES and Race/ethnicity in Cancer-Specific Mortality Risk.**

Supplementary Figure 3. Univariate Cox analysis of cancer-specific mortality by race and socioeconomic status (SES). The low-SES group showed higher cancer-specific mortality compared with the high-SES group, with statistically significant differences observed among Non-Hispanic White and Hispanic patients. Values represent the number of patients who passed away for cancer-specific reasons over the total number of patients within each subgroup.

Abbreviations: CI, Confidence Interval; HR, Hazard Ratio; SES, socioeconomic status.
